# Expert Nordic perspectives on the potential of novel inhalers to overcome unmet needs in the management of obstructive lung disease

**DOI:** 10.3402/ecrj.v2.29445

**Published:** 2015-12-16

**Authors:** Anders Løkke, Lars Ahlbeck, Leif Bjermer, Jann Mortensen, Anders Østrem, Iris Pasternack, Guilherme Safioti, Saku Torvinen

**Affiliations:** 1 Department of Respiratory Medicine, Aarhus County Hospital, Aarhus, Denmark; 2 Allergy Centre University Hospital, Linköping and Respiratory System Drug Committee at Region Östergötland, Linköping, Sweden; 3 Department of Respiratory Medicine and Allergology, Institute of Clinical Science, Lund University, Lund, Sweden; 4 Department of Clinical Physiology, Nuclear Medicine and PET, Rigshospitalet, Denmark; 5 Department of Medicine, National Hospital, Torshavn, Faroe Islands; 6 Gransdalen Health Care Centre, Oslo, Norway; 7 Summaryx Ltd, HTA Research, Helsinki, Finland; 8 TEVA Pharmaceuticals, Helsingborg, Sweden; 9 TEVA Pharmaceuticals BV, Amsterdam, the Netherlands

**Keywords:** asthma*, COPD*, obstructive lung disease*, innovation, patient preference*, patient compliance*, metered dose inhaler*, dry powder inhaler*, medication errors*, economic, outcome, choice, price, cost, reimbursement, access, substitution

## Abstract

The effective self-management of obstructive lung disease is dependent upon the patient achieving good inhaler technique. However, many current inhalers are complicated to use, which may lead to handling difficulties. These difficulties can cause clinically relevant errors, whereby pharmacotherapy fails to achieve adequate lung deposition and therapeutic effect. In this report, the potential of novel inhaler devices to overcome unmet needs in the management of obstructive lung disease is considered by a panel of Nordic experts. The panel concludes that innovative inhalers can contribute to good disease management and better use of healthcare resources.

In the European Union, chronic respiratory diseases affect over 60 million people (1). The burden of respiratory disease is particularly high within the Nordic countries, with Denmark experiencing amongst the highest rates of chronic obstructive pulmonary disease (COPD) in the world (2). Meanwhile, clinical asthma affects approximately 10% of the population across the region (3, 4).

Despite the availability of effective therapy, many patients with obstructive lung disease fail to achieve their treatment goals. It is estimated that 69,000 disability-adjusted life years (DALYs) are lost per annum due to obstructive lung disease across the Nordic region (5). This considerable clinical burden is reflected in the economic impact, with Norwegian medical costs related to COPD estimated to be 284 euros per patient in 2005 (6). When indirect costs are also considered, annual net costs for Danish patients with COPD amounted to 8,572 euros between 1998 and 2010 (7). The economic impact of COPD falls on both patients and healthcare systems, with sufferers experiencing reduction in earnings by approximately 50% compared to their peers (8). Asthmatics in Denmark have also been shown to receive more welfare, sick leave, and disability benefits than non-asthmatics (9). In Finland, it is estimated that total annual COPD-related costs were between 100 million and 110 million euros between 1996 and 2006; this figure is expected to increase to 166 million euros by 2030 (10). In Sweden, a study showed that in 2011, the yearly costs per patient with asthma/COPD were 3,754 euros for primary healthcare centres with an asthma/COPD clinic and 5,930 euros for those healthcare centres without an asthma/COPD clinic. This study also observed that the structured management of patients with COPD increased during the 11-year study period (11).

Both in terms of clinical and economic outcomes, exacerbations are key drivers of burden in obstructive lung disease (12). Consequently, asthma and COPD management seeks to manage and control the disease while preventing the occurrence of severe exacerbations (13, 14). Global clinical guidelines issued by the Global Initiative for Asthma (GINA) and the Global Initiative for Chronic Obstructive Lung Disease (GOLD) recommend that patients at high risk of exacerbations be treated with a fixed-dose combination (FDC) of an inhaled corticosteroid (ICS) and a long-acting beta agonist (LABA), administered via a single inhaler (13, 14).

Inhaler devices can be divided into four classes: nebulisers, soft mist inhalers, pressurised metered dose inhalers (pMDI), and dry powder inhalers (DPI). Within and between these categories, different devices offer different benefits and drawbacks, for example, in terms of ease of use (15). If a patient performs an error, either by lacking the knowledge of how to use the device or by not using the correct technique, this is likely to have a clinically relevant impact on delivery of medication. This is termed a critical inhaler handling error (16). In prescribing inhaler-based therapy, the European Respiratory Society (ERS) considers that a patient's ability to use their device correctly should be a key consideration (15). This recommendation is supported by studies showing the importance of effective inhaler use, with device misuse negatively affecting disease control in up to 90% of patients (17). Conversely, studies suggest that educational initiatives to improve self-management of asthma are associated with improved lung function and reduced emergency room visits (18, 19).

This paper will postulate that inhaler devices represent an important area of unmet needs in the management of obstructive lung disease and that innovative inhalers can contribute to optimal disease management and better use of healthcare resources.

## Methods

A two-stage process was utilised to understand the clinical relevance of inhaler device selection in the treatment of asthma and COPD.

### Semi-structured literature review

Prior to the panel review, a semi-structured literature review was conducted to understand the scope and issues associated with various aspects of obstructive lung disease therapy. Broad search terms were applied to establish a full review of the (English language) literature using PubMed. The terms used were: (asthma OR COPD) AND (inhaler) AND (adherence OR compliance OR persistence OR patient OR preference OR choice OR economics OR social OR outcome). After conducting an initial search, which identified 1,531 articles, a rapid review was conducted to remove 1,103 references (either not related directly to inhalation therapy for asthma and/or COPD, duplicate references/corrections, publications on highly specific/atypical patient populations, or commentary publications on other studies). A final shortlist of 428 papers was used to create materials taken forward for the Nordic Delphi panel review and shared with the expert attendees.

### Delphi-based panel review

A face-to-face panel was convened consisting of six experts from across the Nordic countries (Denmark, Finland, Norway, and Sweden). The goals of the panel were to comment on the existing evidence regarding the clinical relevance of unmet needs related to inhaler devices in the treatment of asthma and COPD.

All experts that participated in the face-to-face panel are the authors of this review. The panel was presented with summary statements of the evidence on a number of critical dimensions identified during the semi-structured literature review. These summary statements and critical dimensions had been prepared by TEVA ahead of the Delphi panel meeting. These were categorised as follows: ‘usage and adherence’; ‘optimisation of the inhaler’; ‘patient preference and choice’; ‘economic impact’; and ‘pricing, value and innovation’.

A semi-anonymous evaluation was conducted on each of the summary statements using an approach based upon the Delphi process. The panel were first presented with a short overview of the evidence base and asked to comment anonymously on the summary statement (in terms of language, importance, and strength of supporting data) and to recommend, again anonymously, enhancements to the data set and the summary of the data.

For each summary statement, the expert feedback was aggregated; subsequently, between one and three rounds of feedback and iteration were completed to establish a consensus on the summary statement which best reflected the group opinion. This consensus has provided the expert perspectives used throughout this review.

## Results

### Inhaler complexity can lead to incorrect usage which may impact patient outcomes

The achievement and maintenance of disease control in asthma and COPD requires meticulous attention to self-management, including the consistent and proper use of medication (20). Failure to follow inhaler device instructions can lead to errors, whereby deposition of medication to the lungs is reduced or prevented entirely (21). As a result, incorrect inhaler device usage can significantly impact the management of obstructive lung disease (13, 22). In COPD, 40% of patients have been shown to make at least one essential mistake in their inhalation technique (23).

While errors in inhalation technique are observed across the general patient population (21), certain groups face particular challenges in achieving optimal technique. For example, incorrect use of inhaler devices is particularly common in the elderly population and in patients with poor cognitive ability or low inspiratory flow rates (23–25).

**Fig. 1:**
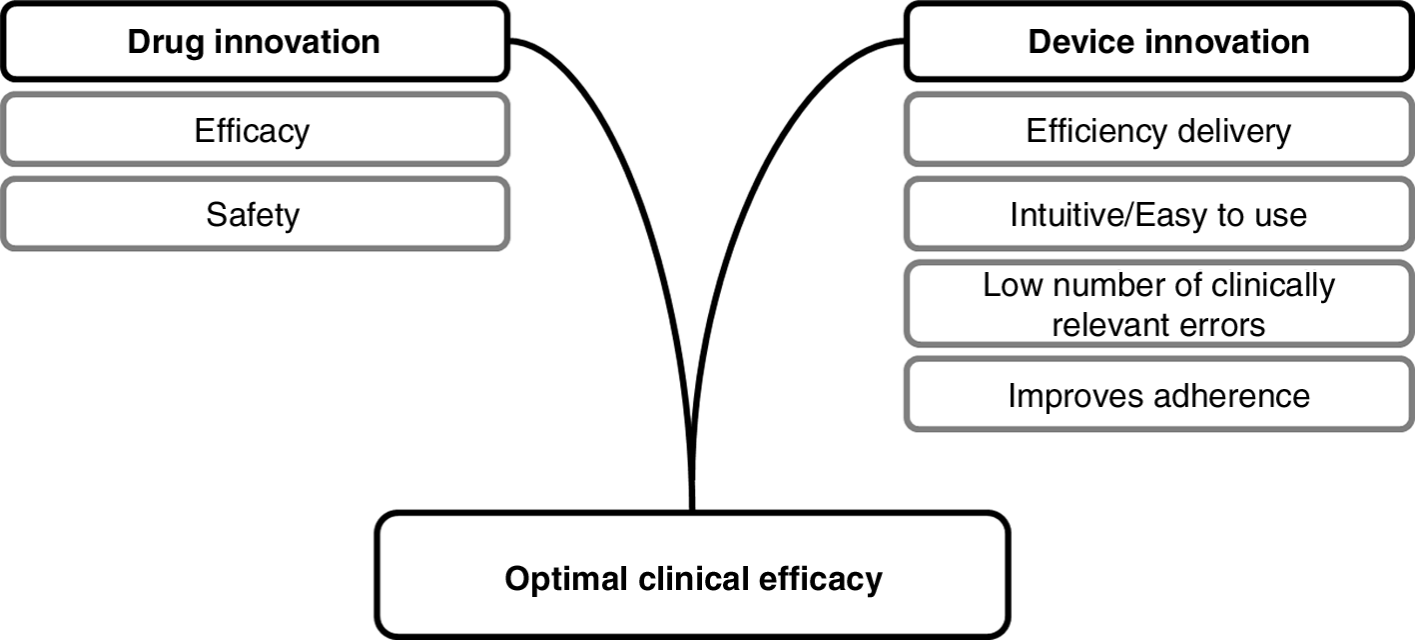
Optimal clinical efficacy is dependent on both drug and device innovations.

The occurrence of clinically relevant errors in inhaler technique has been correlated with reduced levels of disease control in obstructive lung disease, with increased limitations to everyday life, shortness of breath, uncontrolled disease, and sleep disturbance (26). In addition, high levels of clinically relevant inhaler errors were associated with significant increases in unscheduled demands on healthcare resources, including hospitalisation, emergency room visits, and antibiotic or oral corticosteroid use (26, 27). These findings are supported by a French study which demonstrated that asthma patients with good device coordination had significantly better disease control. Data were available from 3,709 asthma patients and showed that those with better inhaler technique also had lower asthma instability index scores (AISs) (17). Misuse of inhaler devices is also an important factor in adverse events. Overdosing due to lack of effect as a result of patient error may impact safety and tolerability (26). Figure 1 represents the key elements supporting optimal clinical efficacy.

Traditionally, pMDI and DPI inhalers often require that patients perform complicated, multistep processes to achieve successful dosing. These procedures can require a considerable degree of dexterity and coordination of inspiration and actuation (28). There have been recent advances in inhaler design focussed on the development of simplified inhalation manoeuvres with enhanced ease of use (29). However, much complexity still remains in the use of current inhaler devices.

### Optimisation of the inhaler device represents an important area of unmet needs in the management of obstructive lung disease

The technical characteristics of the device and drug formulation play an important role in supporting disease control (30, 31). This is driven by the fact that the level of deposition of medication to a patient's lungs is reliant upon the achievement of correct inhaler technique and the avoidance of errors (30). Real-world evidence suggests that disease control is worsened by the occurrence of errors in inhaler technique (26). This may explain why, in a survey of expert physicians, 66% cited ‘failure to master device’ as a leading reason for lack of efficacy in respiratory disease control (32).

Features of a device which may lead to inconsistent dosing include the need to clean excess powder from the inhaler mouthpiece – without which inconsistent dosing can occur (33). Poor coordination of inhalation and actuation has also been identified as an important feature of incorrect technique when using pMDI devices (34). A number of studies have also highlighted the lack of a feedback function in some inhalers as a factor in incorrect usage. Without feedback, patients are unable to confirm whether dosing has been performed correctly – this means that they cannot judge whether a repeated attempt at dosing is necessary (35). Feedback mechanisms can include sounds, tastes, lock systems, and dose counters, with each of these providing different benefits.

The fact that existing inhaler devices are often difficult and non-intuitive to use complicates patient education and disease management and reduces healthcare efficiency (36). Furthermore, inhalation technique may deteriorate over time, and some patients fail to achieve good device handling even after repeated training by a healthcare professional (37).

### Consideration of patient characteristics and their preferences over the choice of inhaler can improve adherence

Non-optimal inhaler usage can occur when patients fail to initiate treatment or when the patient fails to follow the prescribed regimen (38). While the reasons for suboptimal adherence are multifaceted, GINA has reported that difficulty with inhaler devices is a key factor associated with poor adherence (13). This is supported by studies which have suggested that inhaler ease of use is associated with improved adherence (39, 40). Assessments of inhaler usage in real-life settings have shown that different inhalers are associated with different levels of user friendliness. In one study in 3,811 patients, the rate of clinically relevant errors observed ranged from 11 to 32% depending on the inhaler used (41).

Patients who are able to use their inhaler effectively are more likely to express a preference for this device (42–44). In obstructive lung disease, such patient satisfaction has been shown to correlate with improvements in treatment adherence, disease control, and quality of life (44). Supporting these findings, a real-world observational study in 2,135 asthma patients has demonstrated that increasing patient satisfaction with an inhaler was associated with improved treatment adherence (44).

Use of a patient-preferred inhaler may also support the efficient use of healthcare resources. In 100 patients who were randomised to receive one of seven different devices, it was shown that 14% of asthma costs could be saved by selecting an inhaler based on patient preference (43) and assessed within the limits of what the healthcare provider considers adequate. This finding is supported by studies which suggest that selecting a patient-preferred inhaler can be cost effective, even when the inhaler is more expensive than standard inhalers (27).

As new devices with enhanced usability become available, the role of healthcare professionals will be critical in ensuring that the options which best meet patient needs are selected. As part of this, it is important that treatment choices be modified as patients’ abilities and preferences change (45).

### Innovative inhalers can contribute to good disease management and better use of healthcare resources

Among asthma-treated patients, the proportion assessed as having ‘not well controlled’ disease was 53.5% in 2010 (46). It is estimated that asthmatics with uncontrolled disease cost the healthcare system approximately four times more than those with good control on therapy (47). Innovation in inhaler design has the potential to support tailoring of healthcare to individual patient needs, by providing a greater variety of choice over device characteristics.

An intuitive inhaler, which supports optimal patient usage and patient satisfaction, may have the potential to reduce costs. Preferences have been expressed by patients for inhaler devices which are small, with ergonomic mouthpieces, and an easy-to-use dose preparation mechanism (48). Meanwhile, healthcare providers consider that an ‘ideal’ inhaler would be easy to handle, provide confirmation of successful inhalation, be convenient, and have a size and shape optimised for use (49).

In Sweden, the county of Östergötland recently recommended a DPI inhaler which incorporates a feedback mechanism which confirms to patients whether dosing has been successful. The new device system has received a positive reception from physicians and patients and may have had a positive impact on resource demands. While a link between regional asthma expenditure and the adoption of the new inhaler system cannot be confirmed, Lars Ahlbeck (Convener of the Expert Group on respiratory diseases for Östergötland County Council) has suggested that this may have played a role in levelling off of asthma costs in 2012 and a projected decline in 2013 (35).

### Pricing, value, and innovation

The Delphi panel considered that device innovation can play an important role in supporting optimal patient outcomes. The panel stated that drug innovation and device innovation should ‘play by the same rules’ with regards to healthcare systems’ assessment of their value. It was noted that clear evidence of a link between patient-related outcomes and innovative device usage was required to support assessment agencies in their informed decision making.

## Discussion

This paper has postulated that, despite the availability of highly effective pharmacotherapy, a significant proportion of obstructive lung disease patients are failing to achieve effective management of their conditions. This failure imposes considerable burdens on the patient, through increased exacerbations and reduced quality of life, and on the healthcare system, through increased demand for unscheduled resources.

Inhaler devices remain an area where opportunities for improvement remain. Difficulties in using current devices prevent patients from gaining the maximal benefit from their prescribed pharmacotherapy, reducing healthcare efficiency. Consequently, innovative inhaler development has the potential to bring value to patients and healthcare systems if current limitations can be overcome.

The literature suggests existing inhalers are distinguishable from each other in terms of their impact on patient adherence, patient outcomes, and patient preference. Despite this, healthcare decision makers have tended to view inhaler devices as interchangeable. Although differences between pharmacological agents are recognised and appropriately valued, clinically meaningful differentiation in inhaler devices has often been overlooked. An example of this phenomenon can be seen in the recent *Tandvårds-och Läkemedelsförmånsverket* (TLV) reassessment of combinations of ICSs and long-acting beta-2 agonists in asthma and COPD, where it was stated that ‘Among the inhalation powders, there are primarily two main groups, budesonide in combination with formoterol and fluticasone in combination with salmeterol’ (translated from Swedish) (50). Another example of this tendency was presented by the Norwegian Medicines Agency when it was decided that Airflusal Forspiro and Seretide could not be listed for substitution as this would require an increased need for training of patients in inhalation technique (51). Similarly, although the Danish *Sundhedsstyrelsen's* recommendations recognise that inhaler selection should include a consideration of patient preference, emphasis is given to selection of the cheapest treatment option within each medicinal product group (52). This demonstrates that assessment agencies may tend to view inhaler-based therapy in terms of constituent pharmacotherapy, while overlooking potentially valuable differentiation resulting from inhalation devices. Failure to appreciate the importance of individual device characteristics may lead to non-consented switching. Evidence has shown that non-consented switching of medications and inhalers in patients with asthma can be associated with a range of negative outcomes resulting in increased demand for healthcare services as a result of compromised symptom control and a poorer quality of life for the patient (53).

Over the coming decade, progress in inhaler device technology has the potential to provide value to patients and healthcare providers. Whether this be the incorporation of improved feedback mechanisms, or the inclusion of digital technology, it is likely that inhaler devices will incorporate an increasing number of characteristics which provide benefits quite distinct from the molecules which they deliver. The incorporation of digital technology into inhaler devices may, for example, allow patients and physicians to monitor treatment adherence, receive reminders about dosing, and collect ‘big data’ to support ongoing improvements in targeted care (29, 54). These changes will mean that inhaler devices may sit at the centre of an emerging ‘treatment system’ that enables patients to maximise the value extracted from their prescribed care.

The realisation of this promise will depend upon significant investment from the developers of inhaler devices. However, access procedures which treat inhaler devices as substitutable entities may reduce incentives for investment in device innovation and also prohibit patients from accessing new inhaler technology. Healthcare systems should support and encourage device-led innovation by ensuring that reimbursement decisions are not a barrier to access for devices which provide genuine, clinically relevant benefits for patients.

In summary, innovation in devices can be as important as innovation in drug molecules in supporting optimal patient outcomes and quality of life. Consequently, there is a continued need for investment in device innovation that seeks to overcome unmet needs and improve ease of use.
